# Short-term outcomes of robotic-assisted laparoscopic versus laparoscopic lateral lymph node dissection for advanced lower rectal cancer

**DOI:** 10.1007/s00464-020-07979-6

**Published:** 2020-10-01

**Authors:** Hajime Morohashi, Yoshiyuki Sakamoto, Takuya Miura, Takuji Kagiya, Kenta Ogasawara, Yoshiya Takahashi, Kentaro Sato, Yutaro Hara, Hirokazu Ogasawara, Kenichi Hakamada

**Affiliations:** Department of Gastroenterological Surgery, Hirosaki University Graduate School of Medicine, 5 Zaifu-cho, Hirosaki-shi, Aomori, 036-8562 Japan

**Keywords:** Rectal cancer, Lateral lymph node dissection, Robotic-assisted laparoscopic surgery

## Abstract

**Background:**

Therapeutic strategies to suppress local recurrence, including lateral lymph node metastasis, are important to improve the curability of rectal cancer. The aim of the present study was to clarify the advantages of robotic-assisted laparoscopic lateral lymph node dissection (RALLD), comparing its short-term outcomes with those of laparoscopic lateral lymph node dissection (LLLD). There are some retrospective reports comparing RALLD or LLLD and open lateral lymph node dissection (OLLD), but few reports comparing RALLD and LLND to each other.

**Methods:**

From November 2014 to August 2020, we compared the short-term outcomes in 40 patients who underwent RALLD and 55 patients who underwent LLLD.

**Results:**

The total operative time was significantly longer in the RALLD group than in the LLLD group (*p* < 0.001). However, lateral dissection time was not significantly different between the groups (*p* = 0.661). The postoperative hospital time was shorter in the RALLD group than in the LLLD group (*p* < 0.048). No significant differences were identified in the rates of postoperative bleeding, incisional surgical site infection (SSI), organ/space SSI, urinary disfunction, urinary infection, or small bowel obstruction between the groups. However, anastomotic leakage was significantly lower in the RALLD group than in the LLLD group (*p* = 0.031).

**Conclusions:**

The short-term outcomes of RALLD indicate it is feasible, and RALLD may be a useful modality for lower rectal cancer.

Robotic-assisted laparoscopic surgery has technical advantages over laparoscopic surgery because it provides 3-dimensional visualization, a magnified view, a stable camera platform, and improvements in dexterity in terms of the surgical instruments through multi-joint function [1]. Several studies have reported the advantages of robotic-assisted laparoscopic surgery for rectal procedures [2]. Local recurrence of rectal cancer is associated with a poor prognosis, and a therapeutic strategy to suppress local recurrence, including lateral lymph node metastasis, is important to improve the curability of rectal cancer. Total mesorectal excision (TME) is used to treat rectal cancer in hospitals worldwide [3, 4].

However, TME with preoperative chemoradiotherapy (CRT) is the standard treatment method in Europe and the United States, whereas the conventional treatment method in Japan is TME with lateral lymph node dissection (LLD) [5]. According to a Japanese study, the incidence of lymph node metastasis in 2916 patients with rectal cancer was 20.1%. Among those who underwent LLD, the risk of pelvic recurrence was reduced by 50%, and the 5-year survival rate was expected to improve by 8–9% [6]. Therefore, in high-volume centers in Japan, the recommended standard procedure for advanced lower rectal cancer is TME with LLD. However, LLD is technically difficult because it is performed in the pelvic cavity, which is narrow and anatomically complex. Therefore, it is preferable to perform laparoscopic lateral lymph node dissection (LLLD), which is minimally invasive and provides a magnifying effect; however, there is a concern with LLLD that sufficient dissection may not be possible due to the limited range of arm motion available laparoscopically. It is expected that robotic-assisted laparoscopic lateral lymph node dissection (RALLD), in which the arm moves freely, will be useful for LLD and offer safer and more precise surgery.

There are some retrospective reports comparing RALLD or LLLD and open lateral lymph node dissection (OLLD) [7, 8], but little research has been performed that compares RALLD and LLLD. The present study attempted to clarify the advantages of RALLD by comparing its short-term outcomes with those of LLLD at a single center.

## Materials and methods

### Patients

**Fig. 1:**
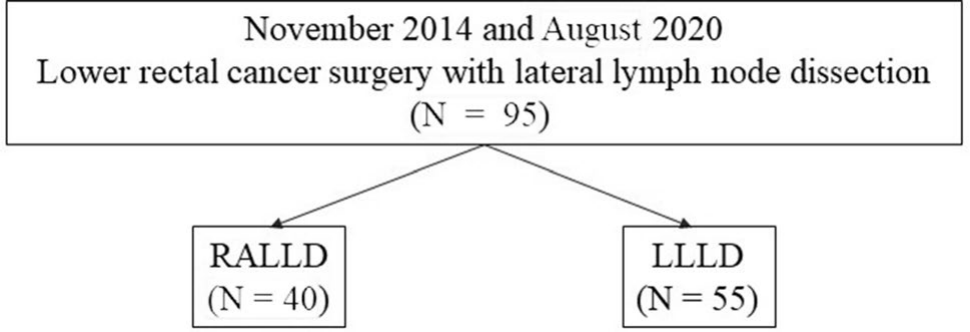
Flowchart of patients. *RALLD* robotic-assisted laparoscopic lateral lymph node dissection. *LLLD* laparoscopic lateral lymph node dissection

Ninety-five patients with lower rectal cancer who underwent LLD following TME with either robot-assisted laparoscopy or a standard laparoscopic procedure at the Department of Gastroenterological Surgery, Hirosaki University, between November 2014 and August 2020. We compared the short-term outcomes in 40 patients who underwent RALLD and 55 patients who underwent LLLD (Fig. 1). The clinicopathological characteristics of patients were determined from the clinical and histopathologic reports, and the tumor features and stages were classified according to the TNM classification system [9]. Tumor progression, size, and position were evaluated using diagnostic imaging (multidetector-row computed tomography, magnetic resonance imaging, and barium enemas). All surgeries were performed by two trained surgeons with more than 10 years of experience in laparoscopic colorectal surgery. Total operative time and time spent performing LLD were calculated based on videos recorded during surgery. The study protocol was approved by the institutional Ethic Committee of Hirosaki University Hospital (No. 2019-1060). A written informed consent was obtained from each patient before enrollment.

### Treatment strategy

LLD was indicated when the lower margin of the tumor was located below the peritoneal reflection and the tumor invaded the serosa. Such patients underwent LLD following TME with preoperative neoadjuvant chemotherapy (NAC) or CRT. The indication was determined in accordance with the guidelines of the Japanese Society for Cancer of the Colon [10]. Up to December 2015, rectal cancer surgeries with LLD were performed using the laparoscopic method only. In January 2016, this institution started robotic-assisted surgery using the Da Vinci Si surgical system (Intuitive Surgical, Sunnyvale, CA, USA). In Japan, robotic surgery for rectal cancer was approved to be covered by insurance in April 2018. Since that time, RALLD has been the preferred modality for almost all patients, regardless of the clinical stage or type of operation. We compared previous conventional LLLD cases, retrospectively, with contemporaneous cases in which RALLD, the newer, preferred technique was employed.

### Surgical procedure

**Fig. 2:**
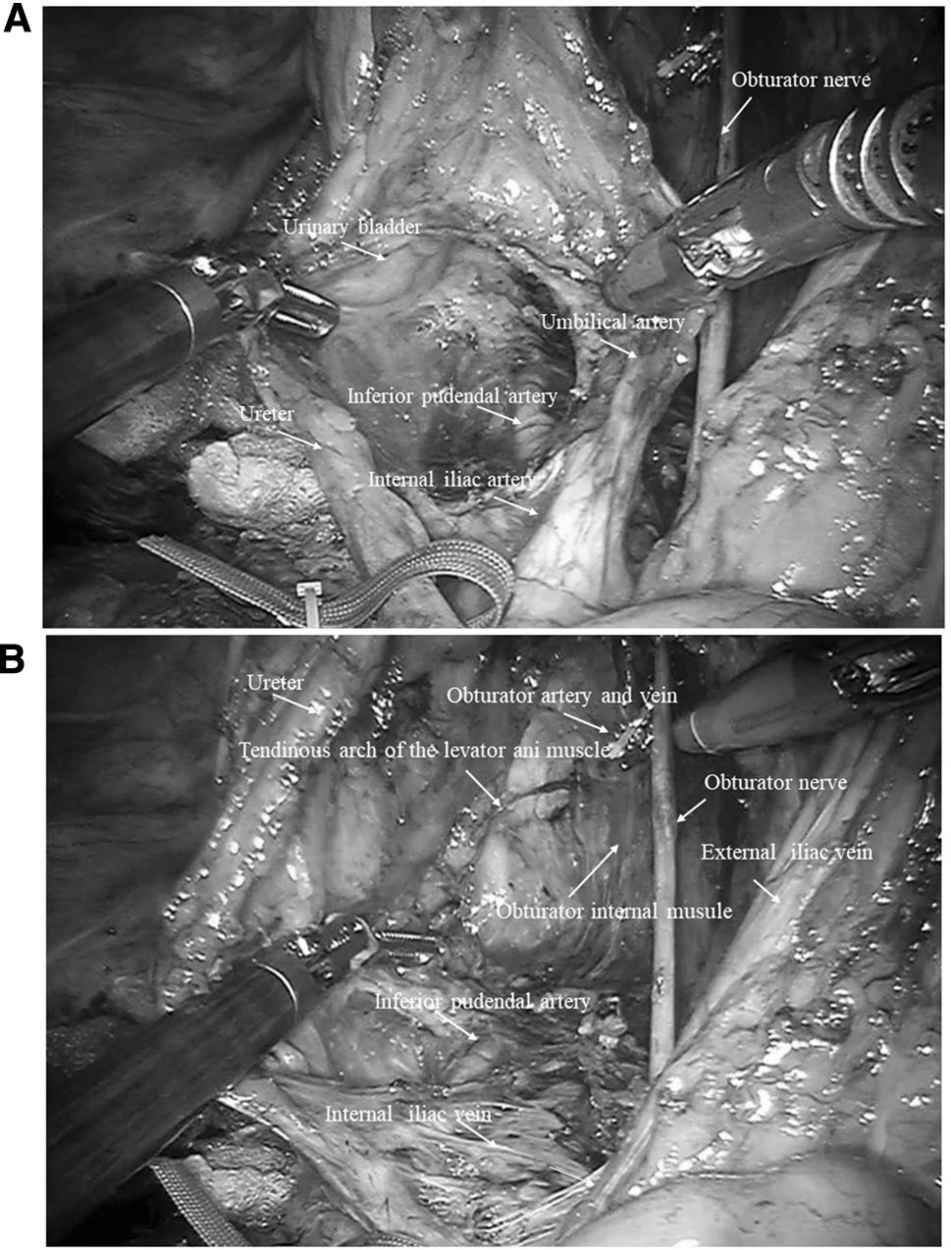
Intraoperative view after right side lateral lymph node dissection of the **a** the internal lymph node area and **b** the obturator lymph node area

All patients underwent bilateral LLD after TME. Proximal lymph node dissection along the lower mesenteric artery was also performed. The location of the LLD was the internal lymph node area and the obturator lymph node area [6]. In brief, the ureter and the hypogastric nerve were isolated from the dissection area to prevent injury. Internal lymph node dissection involved the removal of the fatty tissue on the ventral side of the internal iliac vein and internal iliac artery from the bifurcated cords of the umbilical artery to the lateral urinary bladder (Fig. 2a). The obturator lymph node dissection entailed removal of the fatty tissue from the dorsal side of the external iliac vein to the tendinous arch of the levator ani muscle along the internal obturator muscle (Fig. 2b). The obturator nerve was preserved, while the obturator artery and vein were usually resected. The dissected area for LLLD was the same as that of RALLD.

### Statistical analysis

The Mann–Whitney U test was used to compare continuous variables between the two groups. Categorical variables were presented as patient percentages. *p*-values < 0.05 were considered statistically significant. All statistical analyses were performed using SPSS version 24 (IBM Inc., Armonk, NY).

## Results

### Patient characteristics

Table 1 summarizes the characteristics of the 95 patients in the RALLD and LLLD groups. No significant differences in age and sex were seen between the groups. Other characteristics, such as TNM stage, history of preoperative treatments, and so forth, were not significantly different between the groups. The rate of NAC was higher in the RALLD group than in the LLLD group.

**Table 1:** Characteristics of patients undergoing robotic-assisted or laparoscopic lateral lymph node dissection for rectal cancer

		RALLD (*n* = 40)	LLLD (*n* = 55)	*P*
Age	(years) [median(rage)]	63 (37–75)	63 (34–81)	0.433
Sex				0.473
Male		31 (77.5)	41 (72.7)	
Female		6 (22.5)	14 (27.3)	
BMI	(kg/m^2^) [median(rage)]	22.8 (16.9–32.1)	22.3 (16.0–29.2)	0.610
Tumor location from anal verge	(cm) [median(rage)]	5 (0–8)	5 (0–8)	0.465
cTNM Stage				0.112
I		0	0	
II		24 (60.0)	22 (40.0)	
III		15 (37.5)	28 (50.9)	
IV		1 (2.5)	5 (9.1)	
Preoperative chemoradiotherapy		1 (2.5)	0	0.382
Neoadjuvant chemotherapy		32 (94.1)	43 (78.2)	0.042
History of laparotomy		1 (2.5)	4 (7.3)	0.401

### Perioperative outcomes and postoperative complications

Table 2 summarizes the perioperative outcomes. One patient was converted to laparoscopic surgery in the RALLD group and one patient was converted to open surgery in the LLLD group. The rate of sphincter-preserving procedures, such as low anterior resection or intersphincteric resection, was significantly higher in the RALLD group than in the LLLD group (*p* < 0.001). Total operative time was significantly longer in the RALLD group than in the LLLD group (*p* < 0.001). However, lateral dissection time was not significantly different between groups. There was no significant difference in blood loss between the RALLD group and the LLLD group (*p* = 0.665). The postoperative hospital time was shorter in the RALLD group than in the LLLD group (*p* < 0.048). Table 3 summarizes postoperative complications. No significant differences were identified in the rate of postoperative bleeding, incisional surgical site infection (SSI), organ/space SSI, urinary disfunction, urinary infection, or small bowel obstruction between the groups. However, the rate of anastomotic leakage was significantly lower in the RALLD group than in the LLLD group (*p* = 0.031).

**Table 2:** Comparison of perioperative outcomes between the two groups Values given are numbers (percentages) unless indicated otherwise

	RALLD (*n* = 40)	LLLD (*n* = 55)	*P*
Type of operation			< 0.001
LAR	20 (50.0)	18 (32.7)	
ISR	6 (15.0)	15 (27.3)	
Hartmann's operation	5 (12.5)	3 (5.5)	
APR	9 (22.5)	19 (34.5)	
Bilateral lateral lymph node dissection	33 (97.1)	55 (100)	0.788
Conversion			0.618
Laparoscopy	1 (2.5)	–	
Laparotomy	0	1 (1.8)	
Operative time (min) [median(range)] Total Operative time	507 (270–763)	345 (230–609)	< 0.001
Lateral lymph node dissection time	125 (95—174)	110 (119- 156)	0.661
Blood loss (ml) [median(range)]	60 (0–880)	80 (0–750)	0.665
Transfusion	1 (2.5)	0	0.513
Days to soft diet (days) [median(range)]	3 (3–7)	3 (3–16)	0.401
Postoperative hospital time (days) [median(range)]	14 (10–31)	16 (8–82)	0.048

*RALLD* robotic-assisted lateral lymph node dissection, *LLLD* laparoscopic lateral lymph node dissection

**Table 3:** Comparison of postoperative complications between the two groups

	RALLD (*n* = 40)	LLLD (*n* = 55)	*P*
Incisional surgical site infection	2 (5.0)	3 (5.5)	0.632
organ/space surgical site infection	3 (7.5)	4 (7.3)	0.669
Postoperative bleeding	0	0	–
Small bowel obstruction	0	0	–
Anastomotic leakage	1 (3.8)	7 (21.2)	0.031
Urinary disfunction	4 (10.0)	7 (12.7)	0.667
Urinary infection	1 (2.5)	5 (9.1)	0.132
Obturator nerve paralysis	3 (8.8)	6 (10.1)	0.641

Values given are numbers (percentages) unless indicated otherwise

*RALLD* robotic-assisted lateral lymph node dissection, *LLLD* laparoscopic lateral lymph node dissection

### Pathological outcomes

Table 4 summarizes pathological outcomes. Nine patients in the RALLD group (22.5%) and four patients in the LLLD group (7.3%) exhibited a pathological complete response (*p* = 0.064). There were no significant differences in pathological stages, histological types, tumor sizes, number of dissected lymph nodes, or frequency of positive resected margins. The number of dissected lateral lymph nodes was 25 (3–59) in the RALLD group and 26 (3–62) in the LLLD group (*p* = 0.541). The incidence of lateral lymph node metastasis was 10.0% in the RALLD group and 14.5% in the LLLD group (*p* = 0.251).

**Table 4:** Comparison of pathological results between the groups

	RALLD (*n* = 40)	LLLD (*n* = 55)	*p*
pT			0.064
T0 (pathological complete response)	9 (22.5)	4 (7.3)	
T1	3 (7.5)	2 (3.6)	
T2	10 (25.0)	13 (23.7)	
T3	17 (42.5)	34 (61.8)	
T4	1 (2.9)	2 (3.6)	
pN			0.111
N0	27 (67.5)	32 (58.2)	
N1	7 (17.5)	15 (27.3)	
N2	6 (15.0)	8 (14.5)	
pM			0.351
M0	39 (97.5)	50 (90.9)	
M1	1 (2.5)	5 (9.1)	
pStage			0.284
0 (pathological complete response)	8 (20.0)	4 (7.3)	
I	9 (22.5)	14 (25.5)	
II	9 (22.5)	13 (23.6)	
III	12 (32.5)	19 (34.5)	
IV	1 (2.5)	5 (9.1)	
Lateral lymph node metastasis	4 (10.0)	8 (14.5)	0.251
Histological type			0.338
Well or moderately differentiated	39 (97.5)	53 (96.4)	
Poorly differentiated/mucinous carcinoma/signet ring cell	1 (2.5)	2 (3.6)	
Tumor size (mm) [median (range)]	40 (0–60)	40 (0–80)	0.707
Total lymph node dissection [median (range)]	25 (3–59)	26 (3–62)	0.455
Lateral lymph node dissection [median (range)]	15 (1–32)	13 (2–45)	0.509
Positive distal margin	0	0	–
Distance of distal margin (mm) [median (range)]	20 (5–55)	20 (5–55)	0.331
Positive resection margin	1 (2.5)	1 (1.8)	0.634

Values given are numbers (percentages) unless indicated otherwise

*RALLD* robotic-assisted lateral lymph node dissection, *LLLD* laparoscopic lateral lymph node dissection

## Discussion

In the current study, it should be noted that the actual LLD time was almost the same in the two groups; however, the total operative time was significantly longer in the RALLD group. Robotic-assisted surgery tends to take longer with TME compared to laparoscopic surgery. Factors that increase the operation time of robotic-assisted TME are large tumors, edema due to preoperative treatment, and intraoperative bleeding. In such cases, the view of the operative field is obstructed by bleeding or oozing because of difficulty reaching the target with the suction device in narrow spaces like the pelvis. Simply put, the robotic device has bigger arms than those used in laparoscopic equipment. In our experience, adding an assistant port to insert a suction device to keep the operative field clear makes the operation run smoother and the operating time shorter. Also, robotic surgery may have a role in shortening the learning curve for lower rectal resection compared to laparoscopy or open surgery [11]. Eventually, surgical time may be shortened by accumulating and examining more cases of RALLD.

One of the essential goals of treating rectal cancer is to reduce the local recurrence rate and procedures to this effect are being optimized in various countries. In Japan, TME with autonomic nerve-sparing LLD has been performed for many years [12, 13]. This differs from the therapeutic strategies employed in Europe and the United States, where the standard treatment methods are TME and preoperative CRT. LLD in patients with rectal cancer has been reported to reduce local recurrence rates and increase 5-year survival rates [14]. Conversely, a meta-analysis of 20 studies indicated no improvement in prognosis following lateral dissection, although an increase in urogenital system complications was observed [15, 16]. However, many Japanese surgeons use autonomic nerve preserving LLD techniques in order to prevent such complications [17].

On the other hand, preoperative CRT is reportedly effective in controlling local recurrence, but does not necessarily improvement the prognosis [18]. Furthermore, 66% of patients in one study who were diagnosed with metastasis of the lateral lymph nodes via preoperative imaging examinations and who underwent LLD after CRT still were not cancer-free pathologically [19]. Although no randomized controlled studies have been performed to determine the effect of LLD in patients with rectal cancer suspected of having lateral lymph node metastasis, it appears that CRT is not always sufficient to treat metastatic lymph nodes, as it cannot completely eliminate lateral lymph node metastases. Nevertheless, LLD is the most useful approach for achieving local control in patients with metastatic lateral lymph nodes, whereas preoperative CRT is not necessarily recommended [20].

In the JCOG0212 large-scale clinical trial that mainly targeted patients with clinically negative lateral lymph node metastasis, the local recurrence rate in patients who underwent LLD was significantly lower than that in patients who did not undergo the procedure; LLD was particularly effective in suppressing local recurrence within the lateral pelvis, including lateral lymph nodes [21]. It is necessary to improve treatment strategies to increase curability and to reduce complications in patients with rectal cancer. Laparoscopic surgery can provide a better viewing area and allow magnification of images that capture the complicated anatomic structures in the narrow pelvis. It is expected that robotic-assisted laparoscopic surgery will have even more technical advantages for rectal surgery in narrow regions such as the pelvis. Yamaguchi et al. compared short-term outcomes between RALLD and OLLD and reported that RALLD for rectal cancer resulted in significant decreases in the rate of blood loss, length of postoperative hospitalization, rate of wound infection, rate of small bowel obstruction, rate of anastomotic leakage, and rate of urinary retention [8]. Nagayoshi et al. reported that LLLD was associated with less hemorrhaging, shorter postoperative hospitalization, and a larger number of harvested lateral lymph nodes than OLLD. RALLD and LLLD have been reported to have better short-term outcomes than OLLD. Furthermore, RALLD and LLLD have both been shown to be more useful than OLLD; however, it may prove extremely meaningful to discuss the differences between RALLD and LLLD.

The present study found that the clinicopathological data were similar in the two groups. There was one stage IV patient in the RALLD group and five in the LLLD group. The efficacy of LLD for stage IV is unclear [22], but this study indicates that LLD will be utilized when distant metastases can also be resected. In this study, all stage IV patients had resectable liver or lung metastases and their distant metastatic lesions were resected later. The preference at this institution is NAC over preoperative CRT to avoid harmful events caused by radiation therapy. We are now undertaking a new prospective study evaluating neoadjuvant chemotherapy without CRT for lower rectal cancer (Unique trial No. jRCTs021180033).

The present study found that the rate of anastomotic leakage was lower in the RALLD group than in the LLLD group. RALLD is expected to shorten postoperative hospital stays due to less severe complications, such as anastomotic failure. There are two possible reasons for the reduction of anastomotic leakage. First, it is considered that due to its deep reach, a robotic device can move the rectum safely to the anal canal, which is advantageous for the anastomosis. Consequently, blood flow can be confirmed by the ICG fluorescence method during a robotic operation [23]. In Japan, ICG was first covered by insurance for intestinal blood flow issues in 2018. The introduction of robotic surgery as an official procedure under insurance and the introduction of ICG happened at almost exactly the same time. Since ICG could not be performed under insurance in the retrospective cases of LLLD, it is possible that the degree of anastomotic leakage reported for LLLD in this study may have been lower had the intestinal blood flow been evaluated by ICG. One core issue with robotic surgery is economic sustainability [24]. Since the cost of robotic surgery is higher than laparoscopic surgery, it will become necessary, in the future, to analyze whether both the short-term and long-term results merit the high costs incurred. Furthermore, it is important to devise ways to reduce the costs of such procedures.

Urinary dysfunction was not significantly different in the RALLD group and LLLD group in this study. Urinary dysfunction is mainly caused by autonomic nerve injury during surgery, but in JCOG0212, LLD did not increase the rate of urinary dysfunction or male sexual dysfunction [21, 25]. Autonomic nerve damage may occur from LLD alone, but such damage also may occur from TME alone. Because a circumferential margin of < 1 mm is a risk factor affecting the survival rate [26, 27], injuries to pelvic splanchnic nerves and the inferior hypogastric plexus during surgery may be unavoidable due to the substantial tumor circumferential margin that needs to be maintained to prevent local recurrence. Several studies have reported that robotic-assisted laparoscopic surgery with TME was associated with earlier recovery of normal urinary and sexual function compared to laparoscopic surgery [2, 28]. RALLD may be more useful for autonomic nerve preservation than LLLD because of the magnified views and dexterity of multi-joint function in robotic devices.

Pathological outcomes were not significantly different between the RALLD and LLLD groups. The rate of NAC was higher in the RALLD group than in the LLLD group, so the rate of pathological complete response was higher in the RALLD group. No significant differences between RALLD and LLLD were observed in terms of the number of lymph node dissections or the resection margin rates. It is thought that the methods can be considered equal in terms of curability.

The present study had several limitations. First, this study was a single-institution retrospective study. The patient population was quite small. Therefore, additional prospective controlled studies are warranted comparing robotic-assisted laparoscopic and laparoscopic LLD to validate the efficacy and safety of RALLD.

## Conclusions

In conclusion, the present study clarified the safety and technical feasibility of RALLD compared to LLLD. The short-term outcomes of RALLD make it feasible, so RALLD may be considered a useful modality for lower rectal cancer.
